# Encysted hydrocele of the canal of Nuck in an 11-month-old child with a past history of duodenal atresia and Arnold-Chiari malformation: A case report

**DOI:** 10.1097/MD.0000000000014232

**Published:** 2019-01-25

**Authors:** Zlatan Zvizdic, Emir Milisic, Adisa Chikha, Irmina Sefic, Amra Dzananovic, Semir Vranic

**Affiliations:** a Clinic of Pediatric Surgery, University Clinical Center Sarajevo, Sarajevo, Bosnia and Herzegovina; b Department of Pathology, University Clinical Center Sarajevo, Sarajevo, Bosnia and Herzegovina; c Department of Radiology, University Clinical Center Sarajevo, Sarajevo, Bosnia and Herzegovina; d College of Medicine, Qatar University, Doha, Qatar.

**Keywords:** children, differential diagnosis, hydrocele, surgery, the canal of Nuck

## Abstract

**Rationale:**

Hydrocele of the canal of Nuck is a rare developmental disorder and represents of a homolog of hydrocele of spermatic cord in males. Hydrocele of the canal of Nuck is a very rare cause of inguinal swelling in female infants and children. It results from the failure of obliteration of the distal portion of evaginated parietal peritoneum within the inguinal canal, which forms a sac containing fluid.

**Patient concerns:**

We describe a case of hydrocele of the canal of Nuck in an 11-month-old girl with a past medical history of duodenal atresia and Arnold-Chiari malformation.

**Diagnosis:**

Physical examination and ultrasound revealed a soft, cystic, noncompressible, and non-fluctuant labial mass measuring approximately 5 cm.

**Interventions:**

The patient underwent surgical exploration through a right skin crease incision. The cystic lesion was histologically confirmed to be a non-communicated hydrocele of canal of Nuck.

**Outcomes:**

The child is doing well at 1-year follow-up with no swelling or recurrence on the operated side.

**Lessons:**

Hydrocele of the canal of Nuck is a rare developmental disorder but should be considered in a differential diagnosis in young girls with an inguino-labial swelling.

## 1 Introduction

The inguinal canal in the female transmits the round ligament (*ligamentum teres uteri*), which supports the uterus, and sometimes a finger-like extension of the peritoneum called the canal of Nuck, analogous of the processus vaginalis in males.^[1]^ In most cases, the canal of Nuck disappears without a trace in the first year of life, but sometimes it does not disappear completely causing the formation of an indirect inguinal hernia or hydrocele of the canal of Nuck.^[2]^ The accumulation of fluid in this anatomical structure is called hydrocele/cyst of the canal of Nuck or female hydrocele.

We report a case of a hydrocele of the canal of Nuck in an 11-month-old girl previously surgically treated for duodenal atresia and Arnold-Chiari malformation.

## 2 Case report

**Figure 1:**
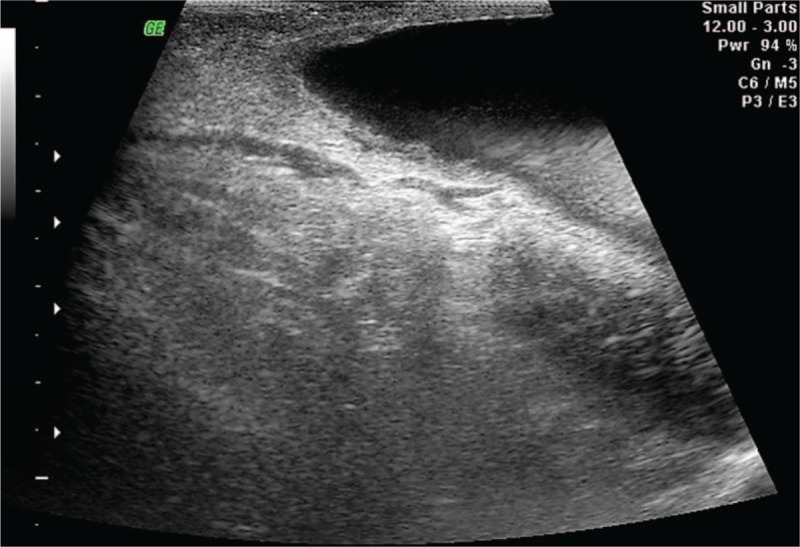
Ultrasonography image of a subcutaneous anechoic, fluid filled mass located near to the right superficial inguinal ring.

An 11-month-old female child presented to our department with a right asymptomatic, painless labial swelling, incipient expressed over the last 3 months and progressively grown in size over the last 2 days. Ultrasonography examination revealed a 4.8 cm × 3.1 cm subcutaneous anechoic, fluid filled mass, located near right superficial inguinal ring without visible connection with the peritoneal cavity (Fig. 1).

**Figure 2:**
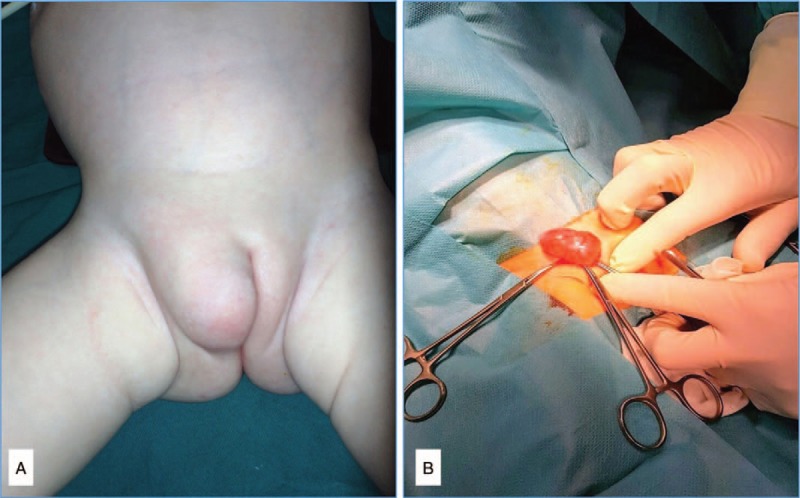
(A) Preoperative presentation of a right-sided inguino-labial swelling and (B) intraoperative view of encysted hydrocele of the canal of Nuck.

Physical examination revealed a soft, cystic, non-compressible, and non-fluctuant labial mass measuring approximately 5 cm × 3 cm (Fig. 2A). Overlying stretched skin was normal. Inguino-labial mass showed a positive transillumination. The remaining physical examination was unremarkable. Defecation and urination were also normal at all times including the last 2 days. Past medical history has shown that the patient has undergone surgical duodenoduodenostomy in the newborn period due to duodenal atresia type 3 and ventriculoperitoneostomy due to hydrocephalus associated with Arnold-Chiari malformation type 1.

Under general anesthesia, the child underwent surgical exploration through a right skin crease incision. The cystic lesion was sent to histopathologic evaluation and tissue dissection; it was confirmed to be a non-communicated hydrocele of canal of Nuck (Fig. 2B). Isolation of the cyst from the round ligament and hydrocelectomy were carried out. The wound was closed in layers using absorbable sutures. Postoperative recovery was uneventful and the child was discharged the next day.

**Figure 3:**
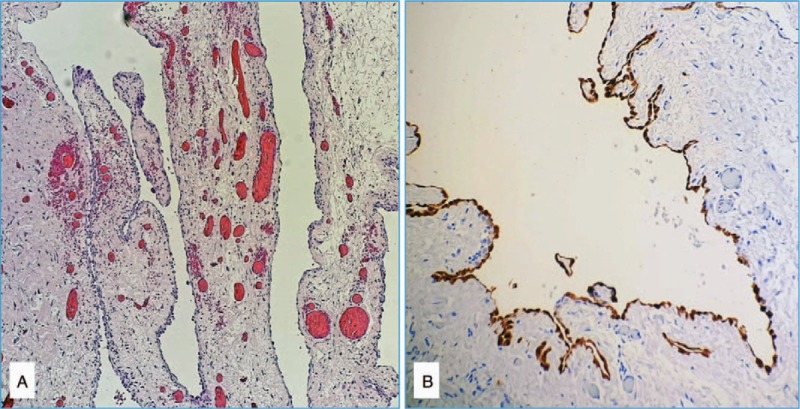
(A) Histopathology showing a cyst wall lined by a single layer of flattened epithelial (mesothelial) cells and containing bundles of smooth muscle fibers (hematoxylin–eosin/H&E/, 10×) and (B) immunohistochemical staining for calretinin confirmed the mesothelial origin of the surface epithelium of the cyst (10×).

Histopathological examination of the specimen confirmed the clinical diagnosis (Fig. 3A and B). The child was doing well at 1-year follow-up with no swelling or recurrence on the operated side.

The study was conducted in accordance with the ethical standards laid down in 1964 Declaration of Helsinki. The case report was shared with the local ethical committee; it is however the policy of the committee not to review case reports. Informed written consent was obtained from the patient for publication of this case report and accompanying images.

## 3 Discussion

A hydrocele of the canal of Nuck is a rare developmental disorder and represents the female equivalent of the processus vaginalis in males. The canal of Nuck usually obliterates within the first 8 months of gestation although its patency may persist postnatally. The canal of Nuck was first described in 1691 by a Dutch anatomist Anton Nuck. There are three types of hydrocele of the canal of Nuck. The most common presentation of hydrocele is type 1, characterized by a unilocular encysted hydrocele, without communication with the peritoneal cavity. A type 2 hydrocele is characterized by a persistent communication between hydrocele and the peritoneal cavity. A type 3 is the rarest form of hydrocele and manifests itself in the form of an hourglass, whereby the internal inguinal ring is compressed by a large hydrocele.^[3]^ In our case, it was a type 1 of unilocular encysted hydrocele without communication with the peritoneal cavity. The differential diagnoses for inguino-labial masses in a female include indirect inguinal hernia, endometriosis of the round ligament, adenopathy, post-traumatic hematoma, and vulvovaginal cyst and tumors (leiomyoma, lipoma, cystic lymphangioma, neuroblastoma metastasis in groin, ganglion cyst, Bartholin's cyst or abscess, and epidermoid cyst).^[1,4,5]^ A hydrocele of the canal of Nuck is a very rare cause of an inguino-labial mass in females, especially in female infants and children and sometimes is overlooked in differential diagnosis for an inguinal mass.^[6]^

The exact cause of cystic formation is unknown, but it is hypothesized that it is caused by the lack of balance of secretion and liquid absorption in the secretory membrane lining.^[7]^ In our case, the girl had ventriculo-peritoneal (VP) shunt due to hydrocephalus before the development of hydrocele of the canal of Nuck. The association between VP shunt and hydroceles or inguinal hernias in male is well recognized,^[8]^ but in females, this association has been rarely reported in the literature. Yuksel et al reported a case of female infant with the appearance of left-side swelling at her left labium majus and groin 5 months after implantation of VP shunt due to lissencephaly and congenital hydrocephalus.^[9]^ They emphasized that VP shunts may clinically present with inguinal manifestations in female infants and may be an important sign of shunt malfunction.^[9]^ In our case, hydrocele of the canal of Nuck occurred 6 months after VP shunt implantation due to hydrocephalus associated with Arnold-Chiari malformation type 1. In contrast to the above-mentioned case where a palpable peritoneal catheter was detected within the cystic mass with shunt malfunction, VP shunt in our patient was functional and the peritoneal catheter did not have any contact with hydrocele of the canal of Nuck. Therefore, it is difficult for us to determine whether the VP shunt could cause a hydrocele of the canal of Nuck or not.

Hydrocele of the canal of Nuck typically presents as a painless, translucent, fluctuating, and non-reducible swelling in the inguinal area and labia majora. Knowing and understanding the clinical presentation of this clinical entity can contribute to the prevention of unnecessary diagnostic tests and enable timely and adequate surgical treatment. In our case, ultrasonography, along with a physical examination finding, was sufficient for a proper preoperative diagnosis of the hydrocele of the canal of Nuck. Open surgical excision of the cyst and ligation of the neck of processus vaginalis proved to be adequate options for the surgical treatment. In addition to the predominant open excision of the hydrocele of the canal of Nuck, laparoscopic excision has been reported in the literature.^[10]^

In conclusion, hydrocele of the canal of Nuck is a rare developmental disorder but should be considered in a differential diagnosis in young girls with an inguino-labial swelling. Ultrasound and physical examination are sufficient diagnostic procedures for the proper clinical diagnosis. However, a surgical exploration and histopathologic confirmation are necessary for the definitive diagnosis.

## Author contributions

**Conceptualization:** Zlatan Zvizdic, Semir Vranic.

**Data curation:** Adisa Chikha, Irmina Sefic, Amra Dzananovic.

**Formal analysis:** Zlatan Zvizdic, Adisa Chikha, Irmina Sefic, Amra Dzananovic, Semir Vranic.

**Investigation:** Zlatan Zvizdic, Emir Milisic.

**Resources:** Semir Vranic.

**Writing – original draft:** Zlatan Zvizdic, Emir Milisic, Adisa Chikha, Irmina Sefic, Amra Dzananovic, Semir Vranic.

Semir Vranic ORCID: 0000-0001-9743-7265.

## References

[1] Anderson CC Broadie TA Mackey JE Hydrocele of the canal of Nuck: ultrasound appearance. Am Surg 1995;61:959–61. 7486426

[2] Manjunatha YC Beeregowda YC Bhaskaran A Hydrocele of the canal of Nuck: imaging findings. Acta Radiol Short Rep 2012;1:10.1258/arsr.2012.110016 PMC3738346 23986837

[3] Sarkar S Panja S Kumar S Hydrocele of the canal of Nuck (female hydrocele): a rare differential for inguino-labial swelling. J Clin Diagn Res 2016;10:PD21–2. 10.7860/JCDR/2016/16710.7284 PMC4800595 27042529

[4] Poenaru D Jacobs DA Kamal I Unusual findings in the inguinal canal: a report of four cases. Pediatric Surg Int 1999;15:515–6. 10.1007/s003830050654 10525914

[5] Pandit SK Rattan KN Budhiraja S Cystic lymphangioma with special reference to rare sites. Indian J Pediatr 2000;67:339–41. 10885205 10.1007/BF02820682

[6] Akkoyun I Kucukosmanoglu I Yalinkilinc E Cyst of the canal of Nuck in pediatric patients. N Am J Med Sci 2013;5:353–6. 23923108 10.4103/1947-2714.114166 PMC3731865

[7] Stickel WH Manner M Female hydrocele (cyst of the canal of Nuck): sonographic appearance of a rare and little-known disorder. J Ultrasound Med 2004;23:429–32. 15055792 10.7863/jum.2004.23.3.429

[8] Celik A Ergün O Arda MS The incidence of inguinal complications after ventriculoperitoneal shunt for hydrocephalus. Childs Nerv Syst 2005;21:44–7. 15071752 10.1007/s00381-004-0954-y

[9] Yuksel KZ Senoglu M Yuksel M Hydrocele of the canal of Nuck as a result of a rare ventriculoperitoneal shunt complication. Pediatr Neurosurg 2006;42:193–6. 16636625 10.1159/000091867

[10] Qureshi NJ Lakshman K Laparoscopic excision of cyst of canal of Nuck. J Minim Access Surg 2014;10:87–9. 24761084 10.4103/0972-9941.129960 PMC3996740

